# HBV-Induced Immune Imbalance in the Development of HCC

**DOI:** 10.3389/fimmu.2019.02048

**Published:** 2019-08-27

**Authors:** Yongyan Chen, Zhigang Tian

**Affiliations:** ^1^Hefei National Laboratory for Physical Sciences at Microscale, The CAS Key Laboratory of Innate Immunity and Chronic Disease, Division of Molecular Medicine, School of Life Sciences, University of Science and Technology of China, Hefei, China; ^2^Institute of Immunology, University of Science and Technology of China, Hefei, China

**Keywords:** HBV, immune dysfunction, HCC, adaptive immunity, innate immunity

## Abstract

Chronic hepatitis B virus (HBV) infection is one of the high-risk factors for human HCC. Despite the integration of virus DNA and the oncoprotein HBx, chronic necroinflammation and hepatocellular regeneration account for hepatocarcinogenesis. As a non-cytopathic virus, HBV is extensively recognized to mediate chronic liver damage through abnormal immune attack. However, the mechanisms driving HBV infection to HCC are poorly understood. During chronic HBV infection in humans, the adaptive immunity changes from immune tolerance to progressive immune activation, inactivation, reactivation and exhaustion, all of which may be the immune pathogenic factors for the development of HCC. Recently, the immunopathogenic mechanisms were described in mouse HBV-induced HCC models, which is absolutely dependent on the presence of HBV-specific T cell response and NK cell-derived IFN-γ, findings which are consistent with the observations from CHB and HCC patients. In this review, we summarize recent research progression on the HBV-specific CD8^+^ T cells, and also CD4^+^ T cells, B cells and non-specific immune cells and molecules underlying chronic HBV infection and eventual HCC development to demonstrate the pathogenesis of HBV-induced immune imbalance. Based on the progression, we discussed the potential of immune-based therapies and their challenges in the treatment of HBV-related HCC, including the checkpoint inhibition, genetically modified T cell transfer, therapeutic vaccines and metabolic modulation.

## Introduction

Chronic hepatitis B virus (HBV) infection is one of the high-risk factors for human HCC, responsible for 50~80% of HCC cases worldwide. As one of the leading causes of cancer death, HCC represents an important human health problem (1). It has been an urgent issue how chronic HBV infection promotes hepatocarcinogenesis. In adults, HBV infection causes a rapid immune response, typically resulting (more than 95% patients) in life-long immunity with acute self-limited infection; while in infants and children, HBV infection has the potential to become chronic with life-long HBV persistence (2).

Despite the direct gene activation and transactivation by the integration of HBV DNA into hepatocyte genome and the oncoprotein HBx and preS/S, HBV-inflicted DNA damage due to hepatocellular regeneration associated with chronic necroinflammation accounts for hepatocarcinogenesis (3–6). In the cytoplasm of infected hepatocytes, HBV has its own nucleocapsids in which HBV completes its replication as a stealth virus without type I IFN-mediated responses (2). As a non-cytopathic virus, HBV-induced liver damage is extensively recognized to be mediated by abnormal immune attack. A dynamic balance between immune clearance and immune tolerance accounts for the outcome in patients with chronic HBV infection. It has been increasingly accepted that immunopathogenesis plays a critical role in HBV-related HCC development; however, the precise mechanisms by which HBV drives to HCC remain poorly understood. Here, the recent progress was summarized in understanding the adaptive and innate immune responses underlying chronic HBV infection that can lead to HCC development and examine the critical pathogenesis of HBV-induced immune imbalance. Further, the cross-talk and network regulation among these immune cells and their released key factors was discussed. Additionally, we discuss the potential of immune-based therapies and their challenges for HBV-related HCC.

### Immune Response Is Related to Disease Progress During HBV Infection

During self-limited acute HBV infection, the efficient HBV-specific immune response is essential. A vigorous response of CD4^+^ T and CD8^+^ T cells was generated to control and clear HBV. HBV-specific CD8^+^ T cells exhibit anti-viral activity by producing IFN-γ and TNF-α (7, 8) or by directly killing the infected hepatocytes (9–11). B cells are co-stimulated by T cells and subsequently produce antibodies to HBV surface antigen (HBsAg), HBV e antigen (HBeAg) and HBV core antigen (HBcAg). These antibodies act to clear antigens and HBV virus from the circulation, preventing or limiting HBV reinfection (12). In addition, NK cells and NKT cells efficiently control HBV, the activities of which peak earlier than that of HBV-specific T cells (12).

**Figure 1:**
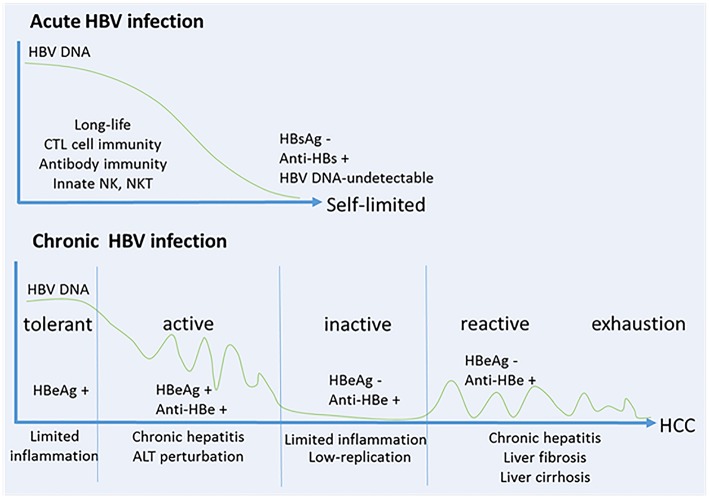
HBV infection relates to the magnitude and quantity of anti-viral immune response. **(A)** Self-limited acute HBV infection. NK cells and NKT cells play important roles in early control of HBV, and then a robust response of CD4^+^ T cells and CD8^+^ T cells is generated to control and eliminate HBV. B cells co-stimulated by T cells produce anti-HBs, anti-HBe and anti-HBc. These protective antibodies clear HBV antigens and virus from the circulation, and prevent or limit HBV reinfection. **(B)** Chronic HBV infection. Five stages are identified including “immune tolerant” stage with a high-replication of HBV-DNA and low-inflammation, “immune active” stage with HBV-specific CD8^+^ T cell response and antibody production which results in chronic liver injuries, inflammations and liver regeneration, “immune inactive” stage with low-replication of HBV and limited inflammation, “immune reactive” stage with chronic hepatitis progressed to liver fibrosis, cirrhosis and HCC, and in the late stage of “immune exhaustion.”

During chronic HBV infection, the early phase termed “immune tolerant” stage with a high-replication of HBV-DNA and low-inflammation during childhood (13). The progressive loss of immune tolerance leads to the “immune active” stage with HBV-specific CD8^+^ T cell responses during adolescence, which results in chronic liver injuries, inflammation and liver regeneration. Patients may subsequently enter an “immune inactive” stage with low level of HBV replication and limited inflammation. Particularly, approximately 20~30% of patients in the inactive carrier stage are subject to a viral relapse, displaying replicative HBV and thus enter the “immune reactive” stage with chronic hepatitis that progress to liver fibrosis, cirrhosis and HCC. In the late stage, a series of oncogenic signaling pathways activated by HBV result in immune escape, and promotes the finally developing HCC (14). More recently, studies show that HBV-immunotolerant patients develop HCC (~12% in 10 years), while treated “immune active” patients develop HCC (~6% in 10 years) with a lower rate. Notably, patients with more cumulative immune-mediated hepatocyte damage would be more susceptible to HCC (15, 16). The patient consequences of HBV infection relate to the magnitude and quantity of anti-HBV immune responses (Figure 1). As one of the hallmarks of cancer, chronic inflammation is considered to be an important element and contributes to changing the tumor microenvironment during this process (17).

### HBV-Specific CD8^+^ T Cell Response in HBV-Related HCC

**Figure 2:**
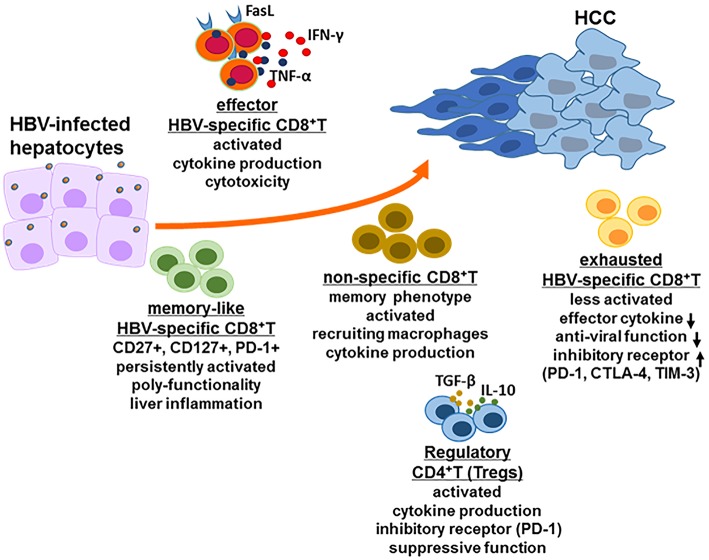
CD8^+^ T cell response in HBV-related HCC. In the HBV-infected individuals, there are several kinds of CD8^+^ T cell populations including exhausted HBV-specific CD8^+^ T cells, HBV core-specific and polymerase-specific CD8^+^ T cells with long-term memory-like phenotype, effector CD8^+^ T cells and HBV non-specific CD8^+^ T cells. The function of each CD8^+^ T cell population is different but possibly promote the development of HCC together. Additionally, regulatory CD4^+^ T cells (Tregs) also play a critical role in the stage of HCC for immune escape, for example inducing CD8^+^ T cell exhaustion.

During acute HBV infection, potent HBV-specific CD8^+^ T cell responses control HBV, typically reducing its titer to undetectable levels. Furthermore, HBV-specific memory T cells with an activated phenotype was persistent after resolving acute HBV infection (18). However, during chronic HBV infection, HBV replication levels as ranging from 10^3^ to 10^9^ HBV DNA copies/mL in peripheral blood of patients with chronic HBV, significantly affected HBV-specific CD8^+^ T cell responses. HBV DNA load of 10^7^ copies/ml was suggested as a threshold, below which multiple HBV-specific CD8^+^ T cells, including env-specific, pol-specific and core-specific CD8^+^ T cells are consistently detected in the peripheral blood; while above the threshold of HBV replication level, env-specific CD8^+^ T cells and pol-specific CD8^+^ T cells can occasionally be found (19). The role and function of highly heterogeneous HBV-specific CD8^+^ T cell are diverse in the development of HBV-related HCC (Figure 2).

The decrease of HBV-specific CD8^+^ T cell functions in CHB patients is mainly shown by the low frequency due to antigen-specific deletion and restricted proliferation, and also the high expression levels of inhibitory receptors such as CTLA-4, PD-1, and TIM-3 (12, 20–23). The higher expression levels of TIM-3 in active CHB patients compared to inactive CHB patients suggest that CD8^+^TIM-3^+^ T cells are functionally exhausted during the active state of chronic HBV infection (24). Oncofetal gene SALL4 reactivation by HBV-induced STAT3 signal in adulthood counteracts miR-200c, which accounted for PD-L1-induced CD8^+^ T cell exhaustion (25). Exhausted CD8^+^ T cells were unable to effectively produce cytokines and exert anti-viral activity when re-exposure to HBV in an acute immune active state, indicating that exhausted CD8^+^ T cells displaying dysfunctional differentiation are associated with chronic HBV replication and the resulting disease progression (26). Furthermore, CD8^+^ resident memory T cells (TRM) were enriched with higher expression levels of PD-1 in the tumor tissue of HBV-related HCC, which were functionally more suppressive and exhausted (27). Single-cell RNA-sequencing demonstrated that there was higher frequency of exhausted CD8^+^ T cells and regulatory T cells (Tregs) with clonally expansion in HCC patients, and layilin (LAYN) was related to the suppressive function of Tregs and exhausted CD8^+^ T cells in HCC (28).

In addition to the inhibitory pathways, a more complex mechanism of energetic and metabolic impairment accounted for CD8^+^ T cell exhaustion during HBV infection. In patients with chronic HBV infection, exhausted HBV-specific CD8^+^ T cells with high ROS level showed extensive downregulation of mitochondrial functions including electron transport, mt DNA transcription and translation, membrane transport and metabolism, and marked downregulation of proteasome subunits and proteins involved in DNA repair (29). Additionally, HBx protein dysregulated glucose metabolism with increased lactate production, which impaired the migration of T cells in the liver and their cytolytic activity (30).

Recently, numerous HBV-specific T cell populations including HBV core-specific and HBV polymerase-specific CD8^+^ T cells were detected in the circulation of CHB patients, and these cells exhibited a long-term memory-like phenotype and poly-functionality, which was not terminally exhausted (31–34). Clinical observation demonstrated that when residual antigen-specific CD8^+^ T cells were persistently activated but unable to control HBV replication, they might contribute to sustain liver inflammations predisposing patients to HCC development (11, 13, 15, 16, 19).

To reveal the exact immunopathogenic mechanisms of CD8^+^ T cells in HBV-related HCC, murine HBV-induced HCC models were generated for analysis. In 1998, Nakamoto et al. (35, 36) demonstrated that HBsAg-specific cytotoxic T lymphocytes (CTLs) constantly attacked HBsAg-expressing hepatocytes, eventually triggering HCC in HBV transgenic mice *via* thymectomy, bone marrow reconstruction and adoptive transfer of splenic HBsAg-specific CD8^+^ T cells from HBsAg-immunized mice. Using this model, they further demonstrated that use of an anti-FasL neutralizing antibody could attenuate the hepatotoxicity of HBsAg-specific CTLs and prevented the chronic hepatitis and eventual HCC (36). Studies in our lab have also illustrated that breakdown of adaptive immune tolerance by blockade of TIGIT (T cell immunoglobulin and ITIM domains, a checkpoint receptor involved in mediating T cell exhaustion in tumors) combined with HBsAg vaccination is able to recover the anti-HBV function of autologous HBsAg-specific CTLs including IFN-γ and TNF-α prodction, which was responsible for mediating HCC progression in HBs-Tg mice (37). To mimick naturally occurring anti-HBV immunity and immunopathology, we generated a novel HBV mouse model by transferring HBsAg^+^ hepatocytes from HBs-Tg mice into an immunocompetent recipient mouse (Fah^−/−^ mouse) with the same genetic background. In this mouse model, HBsAg-specific CD8^+^ T cells were naturally generated and responsible for mediating hepatocyte apoptosis and chronic hepatitis, eventually leading to HCC (unpublished data).

Additionally, non-specific CD8^+^ T cells with memory phenotypes secreted IFN-γ when activated by anti-CD137 mAb in HBV transgenic mice, and played a central role in the subsequent development of chronic inflammation, fibrosis, cirrhosis and HCC progression. During this process, non-specific CD8^+^ T cells preferentially recruited hepatic macrophages, which promoted the development of HCC through secreting TNF-α, IL-6, and MCP-1 (38). In patients with chronic HBV infection, circulating CD14^+^ monocytes with elevated expression of the natural ligand of CD137 might contribute to the sustained CD137 stimulation of CD8^+^ T cells for the liver immunopathology (38).

### HBV-Specific CD4^+^ T Cell Response in HBV-Related HCC

CD4^+^ T cells are considered to contribute to anti-viral and anti-tumor immune responses by producing cytokines that activate CD8^+^ T cells and B cells. Patient circulating and liver-infiltrating CD4^+^ CTLs were increased in the early stage of HCC, which was significantly higher than that of CHB patients (39). This finding indicated that chronic HBV infection may not be the principal element accounting for the observed increase in CD4^+^ CTLs in HBV-related HCC. Both CD4^+^ CTL number and activity decreased in progressive stages of HCC due to the increased Tregs, and the progressive deficit in CD4^+^ CTLs was linked to the high recurrence and poor survival of HCC patients (39).

Tregs are known to exert their suppressive function via cell-to-cell contact or through cytokines such as IL-2, IL-10, TGF-β, and IL-35 (40). Noticeably, in HBV-related HCC patients, Tregs were enriched and showed greater expression of PD-1 with increased suppressive function, which accounted for the more immunosuppressive and exhausted microenvironment of HBV-related HCC compared to the non-virus-related HCC (27). Increased Tregs in HBV-related HCC patients have also been implicated in the reduction of the function of CD8^+^ T cells, as demonstrated by the inhibited proliferation and activation of CD8^+^ T cells and attenuated cytotoxicity of CD8^+^ T cells with less production of granzymeA/B and perforin (41). Persistent presence of HBV led to elevated TGF-β which suppressed miR-34a expression and enhanced CCL22 expression, thus recruiting Tregs in the liver tissue (42). Tregs facilitated the immune escape of HBV^+^HCC, resulting in the development of portal vein tumor thrombus in HCC patients (42). The increased Tregs not only suppressed HBV antigen-specific immune responses, but also suppressed HCC tumor antigen-specific immune responses (43). Further, it was found that compared with the healthy donors and patients of chronic HBV infection, the frequency of circulating CD4^+^CD25^+^CD127^−^ Tregs was much lower in HCC patients, but surgery resulted in significantly increasing the frequency of circulating CD4^+^CD25^+^CD127^−^ Tregs in HCC patients, correlating with tumor aggressiveness (44). These results suggest a therapy targeted to reduce Treg activity may prove beneficial for HCC patients (45).

The frequency of circulating CD4^+^ T follicular helper cells (CXCR5^+^CD4^+^ Tfh) decreased and their function was impaired with disease progression in HBV-related HCC patients (46). Further, the infiltrated CXCR5^+^CD4^+^ T cells was demonstrated to be significantly less in HCC tumor regions than that of non-tumor regions (46). These Tfh cells from HCC patients resulted in less effective induction of the differentiation of plasmablasts from naive B cells, since they reduced ICOS expression and the ability to produce IL-10 and IL-21 with less proliferation activity (47).

### HBV-Specific B Cell and Antibody Responses in HBV-Related HCC

In patients of HBV infection, serological biomarkers changed from HBsAg^+^ and HBeAg^+^ to anti-HBs^+^ and anti-HBe^+^ are used to describe the recovery of HBV infection. HBV is never completely eliminated from the patients system, due to the persistence of a little of covalently closed circular DNA (cccDNA) and integrated DNA of HBV in infected individuals (18). The integrated DNA can express HBV antigens including HBsAg. It is increasingly recognized that humoral immune responses involving anti-HBs antibody production exhibit an important activity in the process of controlling HBV (48, 49). Lack of effective B cell and neutralizing antibody responses promote the disease progression of chronic HBV infection (48). Recently, fluorochromes-labled HBsAg was utilized as “baits” to specifically detect HBsAg-specific B cells *in vitro* by using a dual-staining method. Studies by using this method demonstrated that there was no significant difference in the number of circulating HBsAg-specific B cells among patients with chronic HBV infection, patients with acute HBV infection and vaccinated subjects. Furthermore, the frequency of HBsAg-specific B cells did not correlate to the quantity of HBsAg or HBV-DNA during chronic HBV infection (50). B cells from CHB patients exhibit an atypical phenotype (CD21^−^ CD27^−^) and show functional alterations with PD-1 expression, resulting in the reduction of antibody production (50, 51). When supplemented with cytokine (IL-2 and IL-21) signals and costimulatory signals-derived from CD40L-expressing feeder cells, the maturation of these HBsAg-specific B cells from CHB patients could be partially restored; and functional blockade of the PD-1 expressed on these HBsAg-specific B cells could partially rescue their functions (50, 51). Depletion of B cells by antibody treatment reactivated HBV in patients with chronic HBV infection with a high rate to 60%, even in the subjects with resolved infection years earlier, and this reactivation may lead to severe disease (52).

Beyond the production of HBV-specific antibodies, significantly higher frequencies of IL-10-expressing B cells (Bregs) were observed in HCC patients than that of healthy controls. Furthermore, these Bregs preferentially expressed TIM-1, negatively correlating with the expression of granzyme A/B and perforin in CD4^+^ T cells (53). The different immune phases of patients with chronic HBV infection changes in the Breg frequency, with elevated serum levels of IL-10 observed in the immune active patients when compared to the immune tolerant patients (54). Additionally, the frequency of circulating Bregs was significantly increased after surgery, which was associated with the levels of HBeAg and HBV-DNA copy number (44). These results suggest a potential therapy against Bregs may offer improved outcomes for HCC patients.

### Innate Immune Responses in HBV-Related HCC

The liver as a lymphoid organ has an overwhelming innate immune system, including several kinds of innate immune cells with high frequency such as NK and NKT cells (55–57). Among total intrahepatic lymphocytes, the frequency of NK cells is as high as 25–40 and 10–20% in human and mouse livers, respectively. It is well-known that NK cells exhibit early anti-viral and anti-tumor activities (58); however many mechanisms are involved in the alterations of NK cell functions during the progression of HBV infection.

NK cells participate in controlling HBV replication in mice (59), but within patients with chronic HBV infection their anti-viral activity is suppressed in the presence of IL-10 and transforming growth factor β (TGF-β) (60). Further, in NK cells from patients with chronic HBV infection, inhibitory receptors NKG2A, TIM-3, and PD-1 expression was up-regulated and the ability of IFN-γ and TNF-α secretion was reduced, which was involved in the HCC progression (61–63). Additionally, HBV was reported to secrete exosomes, which play a critical role in mediating HBV transmission in NK cells and consequently impairing NK cell functions, proliferation and survival (61). Furthermore, compared with healthy individuals, NK cells from CHB and HCC patients show significant increases in the expression of microRNA (miR)-146a, which related to the downregulation of NK cell function including reduced cytotoxicity and decreased IFN-γ and TNF-α production (64). In HCC patients, the presence of infiltrating CD11b^−^CD27^−^ NK subsets in the tumor origins was positively correlated with the clinical outcomes, since a substantial proportion of these cells exhibited inactive and immature phenotypes with weak cytotoxicity and poor IFN-γ production (63). In addition to their anti-viral and anti-tumor activities, activated NK cells may also mediate HBV-associated hepatocyte damage (56, 65). Recently, our study demonstrated that NK cells mediate liver inflammation by secreting IFN-γ, which promote the development of HCC through the epithelial cell adhesion molecule (EpCAM)-epithelial cell to mesenchymal transition (EMT) axis in HBs-Tg mice (66). In HCC patients, increased levels of IFN-γ also mediated liver dysfunction and associated with HCC progression (67).

Activation of non-virus-specific cells may result in widespread inflammation, promoting HCC development. NKT cells promoted hepatic stellate cells (HSCs) activation in liver fibrogenesis through producing the inflammatory cytokines IL-4 and IL-13, accounting for the spontaneous liver fibrosis in HBV-Tg mice (68). HSCs upregulated the level of Tregs in the liver, which was involved in the occurrence of HCC following fibrosis and cirrhosis (69). Activation of NKT cells could be augmented by CD205^+^ Kupffer cells through IL-12 production during HBV infection (70). Hepatic macrophages recruited by CD8^+^ T cell-derived IFN-γ subsequently produced TNF-α, IL-6 and MCP-1 to mediate chronic hepatits and HCC progression (38). Circulating CD14^+^ monocytes may contribute to the activation of CD8^+^ T cells through CD137 ligand upregulation in patients with chronic HBV infection (38). Ly6C^+^ monocytes-secreted TNF-α also played the critical role in enhacing CD8^+^ T cell response for HBV clearance (71). Althogh hepatic macrophages promote the development of HCC during chronic HBV infection, they have also shown opposite roles. Kupffer cell-derived IL-10 operates to maintain humoral immune tolerance and induce anti-HBV CD8^+^ T cell exhaustion in chronic HBV carrier (72, 73). Additoanlly, maternal HBeAg-predisposed hepatic macrophages may mediate immune tolerance to HBV (74).

As an important population of liver-resident innate immune cells, γδT cells-drived myeloid-derived suppressor cell (MDSC) accumulation in the HBV-tolerant liver strongly suppressed CD8^+^ T cell function and promoted systemic CD8^+^ T cell exhaustion (75). Recent stuidies demonstrated the circulating γδT cells showed distinct phenotypes and functions with higher frequency of T-bet^hi^ Emos^dim^ Vδ2^+^ γδT cells in patients of chronic HBV infection compared with uninfected control subjects (76). Further, IFNγ/TNF responses were weaker in Vδ2^+^ γδT cells from chronic HBV infected patients with hepatitis flare when compared to those without hepatitis flare (76).

Complement system is a vital part of the innate immune system, comprising a variety of different proteins including complements, complement receptors and the regulatory protein. In CHB patients, elevated C5a promoted HSC activation and inhibited HSC apoptosis, which positively correlated with disease severity demonstrated by the clinical parameters of liver fibrosis (77). Gene polymorphisms of complement receptor 1 (CR1) contributed to the risk of HBV-related HCC in males (78). Additionally, HBx increased the expression of complement regulatory protein CD59, which prevented the formation of terminal membrane attack complex C5b-9 on the hepatoma cells (79). Thus, the dysregulation of complement system induced by chronic HBV infection promoted the development of HCC in several manners.

### Cross-Talk and Regulation Among Different Immune Cells in HBV-Related HCC

**Figure 3:**
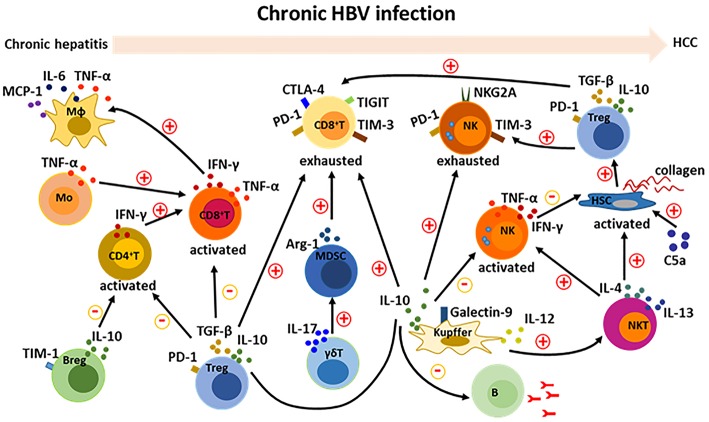
Cross-talk among immune cells in the development of HBV-related HCC. During chronic HBV infection, there are complicate interactions among immune cells. Activation of CD8^+^ T cells is enhanced by CD4^+^ T and monocytes; and then activated CD8^+^ T cells recruit and activate macrophages. Activation of NKT cells can be promoted by Kupffer cells, and further activate NK cells and HSCs. On the other hand, suppressive Tregs, Kupffer cells and Bregs inhibit the activation of CD4^+^ T, CD8^+^ T, and NK cells. Furthermore, Tregs, Kupffer cells, and MDSCs contribute to the formation of exhausted CD8^+^ T and NK cells. A variety of cytokines including IFN-γ, TNF-α, IL-6, MCP-1, IL-4, IL-13, IL-12, IL-17, IL-10, and TGF-β are involved in the cross-talk between immune cells. Additionally, complement protein such as C5a positive regulate the activation of HSCs.

HBV-induced immune imbalance leads to the development of HCC as described above. We further summarized the HBV-induced changed immune cells and their released cytokines (Table 1). Complicated cross-talk and regulation among these immune cells further makes it difficult to control and regulate the immune disorders. A variety of cytokines and other molecules are involved in this process (Figure 3). When these immune cells including CD4^+^T, CD8^+^T, NK, NKT, monocytes/macrophages, and HSCs are activated, they participate in mediating the liver inflammation during chronic HBV infection, which eventually promote the development of HCC. Moreover, interactions among these activated cells through producing cytokines such as TNF-α, IFN-γ, IL-12, IL-4, and IL-13 aggravate the chronic hepatitis (38, 68, 70). On the other hand, several kinds of immunosuppressive cells including Treg, Breg, MDSC, and Kupffer cells negatively regulate those activated immune cell especially by producing cytokines such as TGF-β and IL-10 (27, 41, 53, 54, 60, 75). Noticeably, the negative regulation is also a key factor in inducing the exhaustion of CD8^+^ T and NK cells, resulting in the immune escape of HBV and HCC tumor cells (26, 40, 63, 73). Additionally, the protective antibody production of B cells was also inhibited by Kupffer cell-derived IL-10 (72). How to control and maintain the immune balance is a key issue in the treatment of HBV-related HCC.

**Table 1:** HBV-induced changed immune cells and released cytokines.

**Immune cells**	**Released cytokines**	**References**
CD8^+^ T cells	IFN-γ, TNF-α, IL-2, IL-10, IL-17, IL-21	(7, 8, 37, 38, 80–83)
CD4^+^ T cells	IFN-γ, IL-21,IL-17	(39, 84, 85)
NK cells	IFN-γ, TNF-α, IL-10	(12, 66, 86)
NKT cells	IFN-γ, IL-4, IL-13	(12, 68, 70)
Macrophages	IFN-γ, IL-6, MCP-1,IL-1β, TNF-α, CXCL10	(38, 87)
Monocytes	TNF-α, IL-10, TGF-β	(71, 86)
Hepatic stellate cells (HSCs)	IL-1β, IL-6, TGF-β	(85)
γδT cells	IL-17, TNF-α	(75, 88)
Kupffer cells	IL-10, TGF-β, IL-12, IL-6, TNF-α	(70, 72, 73, 89)
Regulatory T cells (Tregs)	IL-10, TGF-β, IL-35	(27, 40, 41)
Regulatory B cells (Bregs)	IL-10	(54, 90)

### Immune-Based Approaches to HBV-Related HCC Therapy

When the disease cycle of inflammation, progressive fibrosis, cirrhosis and regeneration is broken, the pathways to HCC development are interrupted. It has been recognized that immune balance is important to the outcome of HBV infection, and the effective immune responses with sufficient magnitude and quality of the HBV-specific immune cells is able to adequately control HBV (12). Two clinical cases have further confirmed this speculation. HBV can be eliminated from the infected liver in the recipients with pre-existing HBV immunity when transplanted with a liver from the donor of chronic HBV infection (91). Moreover, after bone marrow transplantation, patients with chronic HBV infection develop an effective anti-HBV immune response in their body and clear HBV (92, 93). Based on these increasing understanding of the mechanisms of immune pathogenesis in patients with chronic HBV infection and HBV-associated HCC, immune-based therapies able to restore immunity and consequently eliminate HBV has been proposed and promoted. Such strategies include checkpoint inhibition, genetically modified T cell transfer, therapeutic vaccines and metabolic modulation.

#### Checkpoint Inhibition

In HBV-infected patients, the function of PD-1^+^ HBV-specific CD8^+^ T cells and PD-1^+^ HBV-specific CD4^+^ T cells were suppressed by the expression of inhibitory ligands on several kinds of immune cells including HSCs, liver endothelial sinusoidal cells, liver-resident macrophages and dendritic cells. By using checkpoint inhibitors to block PD-1/PD-L1 function, HBV-specific T cells were reactivated and their functions were improved (21, 22). Furthermore, a combination therapy involving simultaneous stimulation of OX40 and blockade of PD-L1 functionally augmented HBV-specific CD4^+^ T cells to produce IFN-γ and IL-21 (84). HBV-specific B cell response would be promoted by the activated CD4^+^ T cells in patients with chronic HBV infection (50, 51). CTLA-4 and TIM-3 may offer alternative potential immunotherapeutic targets for patients with HBV chronic infection, since these immune inhibitory receptors also correlated to the inactivation of HBV-specific T cells (94). Higher TIGIT expression was observed in the tumor region of HCC patients and was potentially linked to tumor progression. However, prior to the appearance of a tumor in the liver tissue, TIGIT maintains hepatic adaptive immunotolerance and delays tumor initiation (37). Consequently, the distinct roles of TIGIT in the various stages of tumor initiation and progression should be accounted for the use of checkpoint inhibitors.

#### Genetically Modified T Cell Therapy

In HBV-related HCCs, the HBV DNA integration into the host genomes leads to the expression of HBV antigens on HCC cells, which can be presented by specific MHC molecules and subsequently recognized by HBV-specific CD8^+^ T cells. Autologous T cells that are genetically modified to express HBV antigen-specific T cell receptor (TCR) were used to control HBV-related HCC in patients, such as HBsAg-specific TCR-redirected T cells targeted HBsAg^+^ HCC tumor cells and reconstitute anti-tumoral immune responses (95, 96). HLA-A^*^02-restricted HBV envelope- or core-specific TCRs cloned from patients with acute or resolved HBV infection were used to genetically modify T cells with high functional avidity, demonstrating these envelope- or core-specific TCR-transduced T cells could effectively kill hepatoma cells replicating HBV (97). Further work showed that persistently infected hepatocytes carrying HBV cccDNA could be eliminated by redirecting cytolytic T cells against HBsAg-producing cells (98). The first successful clinic trial of HBV-TCR redirected T cell therapy was from a liver transplant patient with extrahepatic HCC metastasis which produced HBsAg due to the HBV DNA integration of tumor cells (96). Since HLA mismatched in the transplant liver, the HBV-TCR redirected T cells only attacked the extrahepatic tumor cells but not the normal hepatocytes. However, in HBV-related HCC patients there are HBsAg^+^ normal hepatocytes and HBsAg^+^ transformed cells, thus HBV-TCR redirected T cells may induce sever liver damage by targeting HBsAg^+^ normal hepatocytes. How to reduce this risk and overcome the drawback deserves further investigation (99). Additionally, non-lytic lymphocytes engineered to express HBV-specific TCR could reduce HBV replication and limit HBV infection by activating apolipoprotein B mRNA editing enzyme, catalytic polypeptide 3 (APOBEC3) (100). These findings suggest that redirecting T cells genetically modified with high functional TCRs may be therapeutic potential in the treatment of CHB and HBV-related HCC.

#### Therapeutic Vaccines

In chronic HBV infection, a therapeutic vaccine against HBV is primarily able to break HBV-specific immune tolerance and elicit an effective immune response. The combination of a therapeutic vaccine (GS-4774) that HBV antigens were engineered to express in yeast and tenofovir disoproxil fumarate (TDF) was shown to improve HBV-specific CD8^+^ T cell responses, strongly with increased cytokine production of IFN-γ, TNF-α and IL-2 to boost the anti-virus immune responses in CHB patients (80). Treatment of GS-4774 resulted in the reduction of Treg numbers in these patients, potentially offering beneficial effects from both single and combination therapy for CHB patients (80).

#### Metabolic Modulation

In tumor microenvironment, the suppression of T cell metabolism by lack of nutrients or accumulation of lactate, lactic acid and kynurenine resulted in the inhibited effector T cell activity and the promoted suppressive Treg cell function (101). Consistent with the progression on the metabolic dysregulation on immune cells by chronic HBV infection, a novel reconstitution therapy by metabolic modulation might be promising for HBV-related HCC. Targeting exhausted CD8^+^ T cells by mitochondrion-targeted antioxidants such as mitoquinone (MitoQ) and piperidine-nitroxide (Mito Tempo) rescued their anti-viral activity demonstrated by significantly enhanced production of IFN-γ and TNF-α, especially the presence of double positive IFN-γ^+^TNF-α^+^ CD8^+^ T cells (29). Similar effects of the metabolic modulation were also observed in CD4^+^ T cells. Regarding the restoration, metabolic modulation by mitochondrion-targeted antioxidants was effective on exhausted T cells in patients with chronic HBV infection, but not on function-competent T cells, which will reduce the risk of indiscriminate T cell amplification and antoimmune reactions *in vivo* when patients were treated. Additionally, the metabolic status of the T cells significantly affects their anti-viral and anti-tumor activity, such as in adoptive transfer of genetically modified T cells. Thus, the effects of immunotherapy may be improved by combination of these two strategies.

## Summary

During chronic HBV infection, the immune imbalance at a cellular and molecular level is highly complex. Although our understanding of HBV immunopathogenesis has improved in recent years, the precise mechanisms governing this disease progression require further investigation. HBV-specific CD8^+^ T cells, HBV-non-specific CD8^+^, CD4^+^T, B, NK/NKT, Kupffer cells, and HSCs are all involved in the development of HBV-related HCC. Furthermore, cell-to-cell interactions and the regulations between these immune cells increase the complexity of HBV immunopathogenesis. Based on these progression, the potential strategies for the intervention and treatment of HBV-related HCC operate to boost the magnitude and quality of the HBV-specific immune responses with the aim of eliminating HBV and maintaining immune homeostasis in patients.

## Author Contributions

YC prepared and wrote the manuscript. ZT directed the content and revised the manuscript.

### Conflict of Interest Statement

The authors declare that the research was conducted in the absence of any commercial or financial relationships that could be construed as a potential conflict of interest.
